# Association of Community Distress With Lung Transplant Waitlist Acceptance

**DOI:** 10.1111/ctr.70289

**Published:** 2025-08-20

**Authors:** William M. Brandon, Alan Jacob, Colin Dunn, Song Zhang, Ang Gao, Fernando Torres, Adrian Lawrence, Irina Timofte, Srinivas Bollineni, Manish Mohanka, Juan Deleija‐Lujano, Adnan Khan, Joseph Crossno, Michael Wait, Matthias Peltz, Christopher Heid, Lynn Huffman, Steve Ring, John Murala, Suresh Keshavamurthy, Alex Jaye Weston, Vaidehi Kaza

**Affiliations:** ^1^ Department of Internal Medicine UT Southwestern Medical Center Dallas Texas USA; ^2^ Peter O'Donnell Jr. School of Public Health UT Southwestern Medical Center Dallas Texas USA; ^3^ Division of Pulmonary and Critical Care Medicine UT Southwestern Medical Center Dallas Texas USA; ^4^ Department of Cardiovascular and Thoracic Surgery UT Southwestern Medical Center Dallas Texas USA

**Keywords:** health disparities, health equity, lung transplantation

## Abstract

**Introduction:**

Access to transplantation is not entirely equitable with several studies demonstrating racial and socioeconomic disparities affecting the transplant process and thereby outcomes. Notably, few studies have focused on disparities prior to waitlisting. This study aimed to characterize the impact of community socioeconomic factors as measured by the Distressed Community Index (DCI) on acceptance for lung transplant waitlisting.

**Methods:**

A retrospective review was performed on 463 patients evaluated for lung transplant waitlisting at our institution between 2016 and 2020. Community distress was calculated using the DCI, which yields a composite Distress Index (cDI) and includes data on various community characteristics. Statistical analysis was done using descriptive statistics and logistic regression methods.

**Results:**

Of the 463 patients included, 333 (71.9%) were accepted and 130 (28.1%) were denied for waitlisting. The mean cDI was 42.5 (±30.0) and 44.8 (±30.8) (*p* = 0.45) for the accepted and declined groups, respectively, indicating mid‐tier distress for both groups by DCI metrics. The cDI was not found to be associated with odds of waitlist acceptance (OR 0.997, CI 0.99–1.004, *p* = 0.455). Furthermore, there was no association between sex, race, ethnicity, public insurance coverage, or any of the subcomponents of the DCI and the odds of successful waitlisting at our institution.

**Conclusion:**

This single‐center retrospective evaluation found that cDI, as calculated by the DCI, and the DCI subcomponents were not associated with transplant waitlist acceptance. Future studies should be done evaluating community‐level socioeconomic disparities and the utility of community disadvantage indexing tools in the lung transplant waitlisting process, with the intentions of conceptually expanding our understanding of the link between transplant outcomes and biopsychosocial candidacy.

## 1 Introduction

Lung transplantation (LTx) is an increasingly utilized treatment option for end‐stage pulmonary diseases, with over 4600 transplants performed each year [1]. Access to LTx, however, has not been entirely equitable, with several studies showing disparities in the areas of gender, race, and socioeconomic status among patients already placed on the LTx waitlist [2, 3, 4, 5, 6]. Socioeconomic status in particular was found to be associated with discrepant access to LTx in patients with interstitial lung disease, with one study showing a 22% increase in the successful transplantation of waitlisted patients per 10 000 dollar increase in median zip code income [7].

While less commonly described, largely due to limitations in data collection, socioeconomic disparities also exist prior to patients successfully being waitlisted for a lung transplant. A 2012 observational study by Quon and colleagues assessed socioeconomic disparities in lung transplant evaluations in cystic fibrosis patients and found that Medicaid insurance, low zip code median income, and not completing high school were associated with decreased odds of being listed for lung transplant [8]. A 2020 case‐control study by Lehr and colleagues built on these findings by further studying access to transplantation in patients with cystic fibrosis and found that patients with fewer socioeconomic barriers to transplant were more likely to be listed for transplant than those with more barriers, regardless of disease severity [9]. Taken together, these studies support the observation that disparities in the LTx process exist prior to waitlisting and should be explored further.

The impact of neighborhood‐level factors on health outcomes has been a recent area of focus in many fields, with the use of readily available index tools. The Area Deprivation Index (ADI) and the Distressed Community Index (DCI) are two tools capable of describing neighborhood‐level poverty used in studies aimed at correlating community factors with clinical processes and outcomes [10, 11, 12, 13, 14]. The DCI is a census‐based mapping tool operated by the bipartisan public policy organization The Economic Innovation Group that describes socioeconomic disparities at the zip code level in the form of a composite Distress Index (cDI) value based on several community features: Percentage of the adult population without a high school diploma or equivalent education, percentage of habitable housing that is unoccupied, percentage of adult population unemployed, percentage of the adult population living below the federal poverty line, median household income as a share of regional median household income, percentage change in the number of jobs, percentage change in the number of regional business establishments [15]. DCI values range from 0 to 100 and can be broken into quintiles by DCI metrics, with 0–20 indicating a prosperous community and 81–100 indicating a distressed community. While both the DCI and ADI offer the ability to quantify neighborhood‐level disadvantage and compare communities at the zip code level, the DCI offers the advantage of giving readily available values for the components that go into its composite scoring. This allows users to compare aggregate disadvantage as well as differences in the subcomponents of the DCI such as median household income and change in business establishments, giving granularity to zip code comparison beyond aggregate disadvantage.

Using the ADI, Goobie et al. found that pulmonary fibrosis patients in the most deprived neighborhoods experienced increased mortality and decreased rates of LTx [16]. Studies using the DCI have shown that patients in the highest quintile of community distress face higher rates of adverse outcomes during heart failure hospitalizations, higher mortality after cardiothoracic surgery, and higher rates of post‐operative complications from various other surgical procedures [10, 11, 12, 13, 14, 17]. Recently, a large cohort study assessing adverse outcomes after peripheral vascular intervention linked higher cDI values to higher mortality rates [17]. Collectively, these studies support the use of index tools in better understanding the role of community‐level disadvantage in clinical research and highlight the value of the DCI in attempting to link zip code level data to clinical outcomes.

The ability of index tools to quantitatively describe community‐level poverty provides the opportunity to characterize inequity in patients undergoing lung transplant evaluation. We aimed to incorporate these tools in assessing pre‐waitlist disparities at our institution by using the DCI to identify neighborhood‐level socioeconomic disparities that might exist in patients evaluated for LTx waitlisting during the years that the DCI data were derived from. We hypothesized that greater DCI values, and therefore greater community distress, would be associated with decreased odds of being accepted for lung transplant waitlisting.

## 2 Methods and Materials

This was a retrospective cohort study at a large, nonprofit academic health system providing care in the North Texas region. After obtaining IRB approval (STU 2023‐0137), we identified adult patients (age >18) that underwent lung transplant evaluation for any indication at our institution between January 1, 2016, and December 31, 2020. All patients had transplant committee decisions documented within this time frame as well. This range of dates was selected in accordance with the years in which the DCI data we used was validated. Patients with duplicate evaluations for the same indication were excluded from the final study cohort. In patients who declined waitlisting, the reason for denial was assessed to identify patients who were declined for waitlisting due to death or being outside of the transplant window (too sick or too healthy to necessitate transplant consideration, assessed at the committee meeting). These patients were thereafter excluded from the study. Those that opted for self‐removal from the evaluation process were also excluded from the final analysis. Patients that had active evaluations without a final decision being made and those with incomplete zip code data were also excluded. Two cohorts were thereafter formed based on whether patients were accepted or declined for transplant waitlisting.

Patient demographic data were collected from electronic health records including age, patient‐reported sex, race, ethnicity, as well as body mass index (BMI), smoking status, and insurance status as explicitly documented. Clinical variables at the time of evaluation for transplant listing were reviewed including indication for transplant as assessed by clinical documentation and baseline spirometry parameters from the most recent pulmonary function testing. These variables were selected as a means of defining the clinical characteristics of our population and mirror the variables used in other studies assessing socioeconomic factors in lung disease and utilize more established databases [3, 4, 5, 16]. The primary reason for being declined was documented from a database of evaluations done at our institution using the direct language from the database and recorded in a standardized fashion as being related to death, self‐removal, too sick, too healthy, psychosocial concerns, weight issues, and multiple comorbidities, with individuals removed from the final cohort as indicated above. At our institution, patients were interviewed by social workers as part of the transplant evaluation process so that psychosocial risks of transplantation may be identified; these risks are evaluated and documented in a standardized fashion using the Stanford Integrated Psychosocial Assessment for Transplant (SIPAT) tool. This tool scores patients in the following four domains in an attempt to numerically risk stratify psychosocial concerns: patient's readiness level, social support system, psychological suitability and psychopathology, and lifestyle and effect of substance use. The SIPAT has shown to be applicable in assessing the psychosocial risk of transplantation across centers [18, 19]. Investigators reviewed clinical documentation by social workers and noted “treatment and follow up adherence concerns”, “support and caregiver concerns”, and “psychological concerns (undertreated psychiatric and substance use disorders)” as the presence of psychosocial risk. Patient five‐digit zip code was collected from the electronic medical record as listed at the time of LTx evaluation. The DCI database was then used to collect values for cDI and the DCI subcomponents for each patient zip code, as documented during the initial evaluation [15]. The DCI was not directly used in the evaluation process, so all DCI data were collected in a retrospective fashion.

Descriptive analysis was performed to summarize categorical variables by counts and proportions, and continuous variables by mean, standard deviation, and median. 95% confidence intervals of estimated parameters were reported. Comparisons between groups were performed using a two‐sample *t*‐test or Chi‐square test. Univariate logistic regression models were constructed to assess the impact of various factors, including cDI, age, sex, race, ethnicity, smoking status, indication for transplant, baseline spirometry, and insurance status. The stepwise variable selection method was employed to obtain the final multivariate model assessing the association of BMI and psychosocial concerns with the outcome of interest given the identified *p* value <0.2 in univariate analysis. This was done in the statistical software SAS. Effects were entered into and removed from the model in such a way that each forward selection step could be followed by one or more backward elimination steps. The stepwise selection process is terminated if no further effect can be added to the model. The cDI was explored either as a continuous variable from 0 to 100 or as a categorical variable as distressed (>60) versus non‐distressed (<60).

## 3 Results

Between 2016 and 2020, our institution received 3207 referrals for lung transplant evaluation, of which 543 underwent evaluation. After exclusion, our final analysis included 463 patients, of which 333 were accepted for lung transplant waitlisting and 130 were declined. The primary reason for waitlisting decline was multiple comorbidities (*n* = 107), the presence of psychosocial concerns (*n* = 22), and weight issues (*n* = 1). Table 1 summarizes baseline characteristics of the accepted and declined cohorts. The demographic makeup of the collective final cohort was 62.6 (±11.0) years of age, 64.5% male, 81.7% white, and 87.8% non‐Hispanic. There were no statistically significant differences in patient‐level demographic or clinical variables between the groups, except for BMI and the presence of psychosocial concerns on social worker assessment. BMI had values of 26.9 (±4.67) versus 25.7 (±4.74) (*p* < 0.0001) for accepted and declined groups, respectively. Psychosocial concerns were present in 50.0% of patients who declined transplant waitlisting and 28.4% in those accepted (*p* < 0.001). The median cDI was 43.1 for the collective cohort. In comparing the cDI between groups, the accepted and declined groups had a mean distress index of 42.5 (±30.0) and 44.8 (±30.8) (*p* = 0.45), respectively. There were 112 (33.6%) patients considered distressed in the accepted group and 48 (36.9%) in the declined group (*p* = 0.50). There was no difference between groups in each individual subcomponent of the DCI (Table 2).

**TABLE 1:** Baseline characteristics of patients evaluated for lung transplant waitlisting.

	Accepted	Declined	All	*p* value
Age (*n* = 463)	62.4 ± 11.1	63.1 ± 10.7	62.6 ± 11.0	0.55^a^
Male sex	218 (65.66%)	80 (61.54%)	298 (64.50%)	0.41
BMI	26.9 ± 4.67	25.7 ± 4.74	26.6 ± 4.71	0.015^a^
Race				0.20
American Indian/Native Hawaiin/Pacific Islander	3 (0.92%)	3 (2.59%)	6 (1.35%)	
Asian	9 (0.75%)	0 (0.00%)	9 (2.03%)	
Black	35 (10.7%)	16 (13.8%)	51 (11.5%)	
Other	12 (3.67%)	3 (2.59%)	15 (3.39%)	
White	268 (82.0%)	94 (81.0%)	362 (81.7%)	
Ethnicity				0.97
Hispanic	40 (12.2%)	14 (12.1%)	54 (12.2%)	
Non‐Hispanic	288 (87.8%)	102 (87.9%)	390 (87.8%)	
Smoking status				0.39
Former	193 (58.0%)	81 (62.3%)	274 (59.2%)	
Never	140 (42.0%)	49 (37.7%)	189 (40.8%)	
Insurance				0.19
Public	37 (11.2%)	20 (15.8%)	57 (12.5%)	
Private	293 (88.8%)	107 (84.3%)	400 (87.5%)	
Presence of psychosocial concerns				<0.001
Absent	234 (71.6%)	56 (50.0%)	290 (66.1%)	
Present	93 (28.4%)	56 (50.0%)	149 (33.9%)	
Primary diagnosis				0.52
Cystic fibrosis	9 (2.70%)	2 (1.54%)	11 (2.38%)	
Obstructive lung disease	56 (16.8%)	29 (22.3%)	85 (18.4%)	
Interstitial lung disease	238 (71.5%)	88 (67.7%)	326 (70.4%)	
Other	16 (4.80%)	4 (3.08%)	20 (4.32%)	
Pulmonary hypertension	14 (4.20%)	7 (5.38%)	21 (4.54%)	
Baseline spirometry				
FVC, % predicted	51.7 ± 18.6	48.7 ± 17.2	50.8 ± 18.3	0.13^a^
FEV_1_, % predicted	47.6 ± 20.7	42.9 ± 19.4	46.3 ± 20.4	0.50^a^
DLCO, % predicted	26.9 ± 13.4	29.9 ± 26.9	27.5 ± 17.3	0.39^a^

^a^ By *t*‐test, otherwise by Chi‐Square Test.

**TABLE 2:** Markers of community distress in patients accepted and declined for transplant waitlisting.

	Accepted	Declined	All	*p* value
Composite Distress Index (*n* = 463)				0.50
<60 (non‐distressed)	221 (66.4%)	82 (63.1%)	303 (65.4%)	
>60 (distressed)	112 (33.6%)	48 (36.9%)	160 (34.6%)	
DCI subcomponents				
No high school diploma (percent of population)	12.5 ± 8.35	13.8 ± 8.56	12.9 ± 8.42	0.13^a^
Poverty rate (percent of population below federal poverty line)	11.8 ± 7.034	12.9 ± 7.95	12.1 ± 7.31	0.17^a^
Adults not working (percent of working population unemployed)	21.4 ± 7.41	21.9 ± 7.47	21.5 ± 7.42	0.47^a^
Housing vacancy rate (percent of population)	8.32 ± 4.91	8.04 ± 4.31	8.24 ± 4.75	0.57^a^
Median household income (thousands of dollars)	70.4 ± 24.7	70.8 ± 33.0	70.6 ± 27.3	0.90^a^
Change in employment (percent change over 5 years)	11.5 ± 43.4	11.6 ± 19.1	11.6 ± 38.2	0.98^a^
Change in establishments (percent change in businesses over 5 years)	9.56 ± 13.5	9.46 ± 14.1	9.54 ± 13.7	0.93^a^

^a^ By *t*‐test, otherwise by Chi‐Square Test.

**FIGURE 1:**
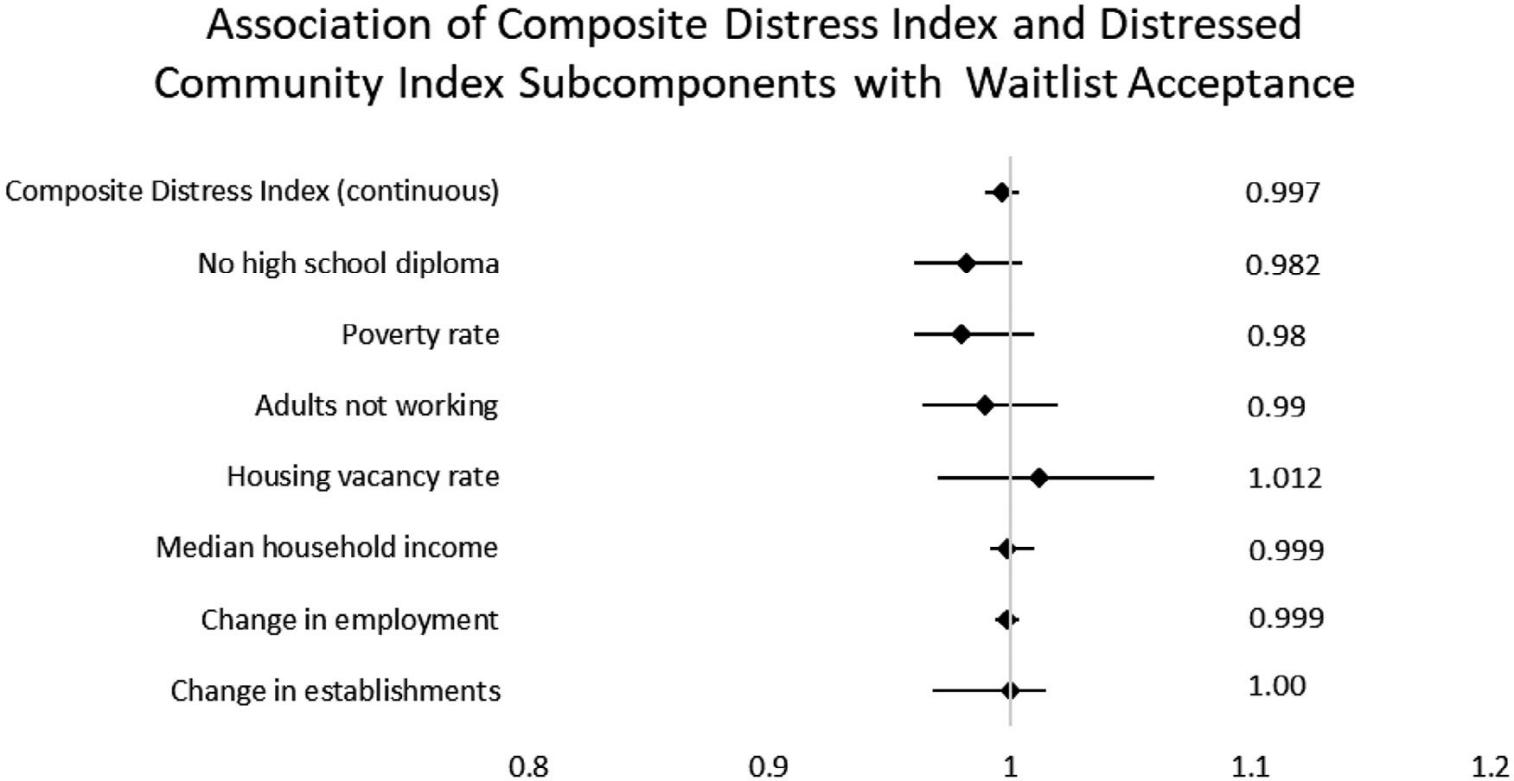
Association of composite distress index and distressed community index subcomponents with transplant waitlist acceptance.

In assessing the relationship between cDI and transplant waitlist acceptance, we found no association when cDI was assessed as either a categorical variable (>60 vs. <60) or a continuous variable (0–100) with analysis showing odds ratios of 0.864 (CI 0.57–1.32, *p* = 0.48) and 0.997 (CI 0.99–1.004, *p* = 0.455), respectively (Table 3). There was no relationship between waitlist acceptance and each of the DCI subcomponents (Tabel 4). Graphical representation of odds ratios of cDI and DCI subcomponents can be seen in Figure 1. There were also no statistically significant associations between odds of transplant waitlist acceptance and sex, race, ethnicity, smoking status, indication for transplant, insurance funding type, or baseline spirometry at our institution (Table 4). BMI had a statistically significant association with waitlist acceptance with an odds ratio of 1.06 (CI 1.01–1.10, *p* < 0.02). Presence of psychosocial concerns was associated with decreased odds of waitlist acceptance with an OR of 0.4 (CI 0.257– 0.62, *p* < 0.0001) (Table 4). Stepwise analysis of the presence of psychosocial concerns and BMI demonstrated an OR of 1.052 (CI 1.003–1.104, *p* < 0.037).

**TABLE 3:** Results of univariate logistic regression analysis of composite distress score and DCI subcomponent on odds of waitlist acceptance.

Variable	Odds ratio	95% C.I. for odds ratio	*p* value
Composite Distress Index (continuous)	0.997	(0.99, 1.004)	0.455
Composite Distress Index ≥ 60 (categorical)	0.864	(0.57, 1.32)	0.48
No high school diploma	0.982	(0.96, 1.005)	0.131
Poverty rate	0.98	(0.96, 1.01)	0.165
Adults not working	0.99	(0.964, 1.02)	0.452
Housing vacancy rate	1.012	(0.97, 1.06)	0.59
Median household income	0.999	(0.992, 1.01)	0.849
Change in employment	0.999	(0.994, 1.004)	0.82
Change in establishments	1.00	(0.986, 1.015)	0.968

**TABLE 4:** Results of univariate logistic regression analysis of patient characteristics on odds of waitlist acceptance.

Variable	Odds ratio	95% C.I. for odds ratio	*p* value
Male sex	1.20	(0.79, 1.82)	0.401
Age	0.995	(0.98, 1.01)	0.572
BMI	1.06	(1.01, 1.10)	0.02
Race (compared to white race)			
Asian	6.69	(0.33, 135.3)	0.216
Black	0.76	(0.40, 1.43)	0.391
Other	1.26	(0.36, 4.37)	0.719
Non‐Hispanic ethnicity	1.008	(0.53, 1.92)	0.981
Never smoker status	1.196	(0.79, 1.81)	0.40
Indication for transplant (compared to cystic fibrosis)			
Obstructive lung disease	0.50	(0.11, 2.31)	0.378
Interstitial lung disease	0.71	(0.162, 3.10)	0.648
Other	0.97	(0.16, 5.87)	0.969
Pulmonary hypertension	0.51	(0.092, 2.82)	0.439
FVC	1.001	(0.997, 1.02)	0.134
DLCO	0.99	(0.977, 1.01)	0.268
FEV1	1.01	(1.00, 1.02)	0.035
Presence of psychosocial concerns	0.4	(0.257, 0.62)	<.0001
Private insurance	1.49	(0.83, 2.68)	0.181

## 4 Discussion

In the present study, we found that in a population of clinically similar patients undergoing LTx evaluation for diverse indications at a large, nonprofit academic health system, cDI, as calculated by the DCI, was not associated with successful transplant waitlisting. In assessing individual components of the DCI, we found no association between high school completion rate, poverty rate, adults not working, median household income, housing vacancy, changes in employment, or changes in establishment and odds of successful waitlisting (Figure 1).

The individual patient characteristics of BMI and presence of psychosocial concerns were statistically associated with odds of waitlisting (increased odds and decreased odds, respectively). However, characteristics such as age, sex, race, and ethnicity had no significant association. It is important to note that BMI and psychosocial concerns are directly used in determining candidacy for transplantation and, therefore, their association with odds of waitlisting does not likely represent a particular inequity in the transplant process as assessed here. Taken in aggregate, our data did not support our hypothesis that higher markers of community distress, as assessed by the DCI, are associated with decreased odds of LTx waitlisting. In considering the implications of these findings at our institution alone, it can be implied that the rigorous selection process by the multidisciplinary team assists in precise decision‐making and may aid in promoting equitable organ allocation. Furthermore, while our findings here did not show evidence of community distress impacting odds of LTx waitlisting or confirm the utility of the DCI in the transplant evaluation process, future investigation should be done in this area with larger, multi‐center populations capable of obviating more subtle statistical differences and confirming our findings across institutions.

Our detailed review of the literature did not yield an extensive amount of research aimed at incorporating the use of an established, publicly available community socioeconomic indexing tool in characterizing disparities prior to LTx waitlisting in a patient population with diverse indications for transplant. Prior studies have been done, however, assessing the role of socioeconomic disadvantage in the lung transplant evaluation process using disease‐specific databases, and have shown that inequity exists prior to LTx waitlisting in certain populations. Two studies assessing pre‐waitlist disparities in a database of patients with cystic fibrosis demonstrated that increased socioeconomic disadvantage was associated with decreased access to the waitlist, regardless of clinical characteristics [8, 9]. Both Quon and colleagues and Lehr and colleagues noted that patients living in zip codes with lower median household income were less likely to be accepted for waitlisting [8, 9], establishing a need to better understand community characteristics in evaluating transplant inequity. It is well‐established that unique features of a patient's living environment impact health in the form of access to food, education, environmental exposures, and behavioral patterns [20, 21, 22, 23, 24, 25], and can lead to increased burden of disease and inequity in healthcare delivery [26, 27, 28, 29].

Our findings do not align with the aforementioned studies that demonstrated that inequities in the lung transplant process begin prior to waitlisting and lead to disparate access to successful waitlisting [8, 9]. We suspect this is largely due to our limited, single‐center study population as compared to other studies in this space that incorporated large, disease‐specific databases, which therefore limited the statistical power behind our observations and minimized our ability to detect meaningful associations. The demographic makeup of our study population was also overwhelmingly white, non‐Hispanic, and male, which are historically factors associated with social advantage [2, 3, 4, 5]. Additionally, our study was composed largely of privately insured patients, further limiting our study population when compared to regions with higher rates of publicly insured patient populations, such as states in which Medicaid has been expanded and includes transplant benefits, and may be more at risk of social disadvantage.

It is worth noting that our assessment of community socioeconomic disadvantage depended on the use of a zip code level indexing tool in the DCI, which requires the application of aggregate data to the study population in an attempt to contextualize socioeconomic realities within the LTx evaluation process. Publicly available indexing tools, such as the DCI and ADI, allow for the geospatial comparison of the socioeconomic environments in which patients live, but do not offer patient‐specific granularity or robust clinical validation and standardization to construct a useful biopsychosocial profile of individual patients that can be integrated into clinical process such as transplant evaluation. While our work here is limited, it attempts to bring attention to the role of neighborhood‐level factors in the LTx evaluation process. We acknowledge that the DCI, as well as the ADI, offers an imperfect means of assessment of a patient's community and the role it plays in clinical processes and outcomes, yet a distinctly more effective tool than the aforementioned ones is yet to be made available. In the future, the development of more targeted and validated markers of community‐level socioeconomic disadvantage and the incorporation of such markers into a new geospatial indexing system may be more effective in linking environmental features to individual patients. Alternatively, the clinical utility of the DCI and ADI should be further explored in large, multi‐center studies that offer the power to identify subtleties in the clinical utility of the DCI and ADI in candidate evaluation and help guide clinicians in assessing candidacy as it relates to socioeconomic equity. Progress in these areas would provide new, clinically useful tools for the study of disparities in LTx, which would ideally lead to more specific and effective interventions for vulnerable patients.

Furthermore, as it relates to multi‐center studies in this area, we acknowledge that the use of select disease‐specific databases has been useful in identifying pre‐waitlist disparities; however, the lack of a large, geographically and clinically diverse source of waitlist evaluation data has limited the ability to generalize findings from previous studies and adequately identify the scope of inequity. Notably, large transplant networks have recently been granted authorization to collect pre‐waitlist data in an effort to promote more equitable access to transplantation, which may remedy this obstacle [30, 31]. We are optimistic that the collection of this data and subsequent development of a pre‐waitlist database will allow for larger, more nationally representative studies to be done in the area of pre‐waitlist disparities. This offers the possibility of creating new means of assessing environmental socioeconomic disparity or validating current indexing tools and integrating the assessment of community disadvantage into the waitlisting process—conceptually expanding our understanding of the link between transplant outcomes and biopsychosocial candidacy. Additionally, future studies assessing health equity in the pre‐waitlisting phase of transplantation including racially and ethnically diverse populations will produce a body of evidence more applicable to a patient population inclusive of historically disadvantaged populations—a well‐defined goal in modern transplantation. This process of disparity characterization and equity‐targeted integration should be conducted in a true multidisciplinary fashion with the inclusion of patients, community stakeholders, clinicians, researchers, and policy makers.

## 5 Conclusion

This single‐center retrospective evaluation found that cDI, as calculated by the DCI, and the DCI subcomponents were not associated with transplant waitlist acceptance. Future studies should be done evaluating community‐level socioeconomic disparities and the utility of community disadvantage indexing tools in the lung transplant waitlisting process, with the intentions of conceptually expanding our understanding of the link between transplant outcomes and biopsychosocial candidacy.

## Conflicts of Interest

The authors declare no conflicts of interest.

## Data Availability

The data that support the findings of this study are available from the corresponding author upon reasonable request.
